# Metabolomic Lipid Profile Changes in Patients with Heart Failure Undergoing Oral Nutritional Supplements Enriched with the Omega-3 (*n*-3) Polyunsaturated Fatty Acids and Mediterranean Diet

**DOI:** 10.3390/nu18132159

**Published:** 2026-07-03

**Authors:** Aura D. Herrera-Martínez, Concepción Muñoz Jiménez, José López Aguilera, Manuel Crespin, María Ángeles Gálvez Moreno, María José Molina Puerta

**Affiliations:** 1 Maimonides Institute for Biomedical Research of Cordoba (IMIBIC), 14004 Córdoba, Spain; 2 Endocrinology and Nutrition Service, Reina Sofia University Hospital, 14004 Córdoba, Spain; 3 Cardiology Service, Reina Sofia University Hospital, 14004 Córdoba, Spain

**Keywords:** heart failure, sarcopenia, oral nutritional supplements, mediterranean diet, lipids

## Abstract

Chronic inflammation and metabolic dysregulation of heart failure (HF) often result in sarcopenia. The combined effect of Mediterranean diet (MD) and omega-3 polyunsaturated fatty acids on the advanced lipidomic profile of HF patients remains poorly defined. Objective: Our objective was to analyze the specific effects of a MD plus omega-3-enriched oral nutritional supplements (MD+ONS) versus MD alone on the metabolic lipid profile of patients with HF, stratified by sarcopenia status. Methods: In this prospective, open-label, randomized controlled trial, 38 patients with HF were assigned to MD alone or MD+ONS (24 weeks). Advanced lipoprotein profiling (triglycerides, cholesterol, particle size, and concentration for VLDL, LDL, and HDL subclasses) was performed using 2D ^1^H-NMR spectroscopy. Results: At baseline, NT-proBNP levels correlated positively with ω6/ω7ω9 fatty acids and IDL-TG (*p* < 0.05). Over 24 weeks, VLDL-C, VLDL-TG, and VLDL-P significantly decreased in the whole cohort (*p* < 0.001). However, stratified analysis revealed that in patients with sarcopenia, these reductions were primarily driven in the MD group (*p* < 0.01). Conversely, in patients without sarcopenia, the MD+ONS group showed significant reductions in VLDL-TG, VLDL-P, and VLDL-Z (*p* < 0.05). Regarding intermediate lipoproteins, IDL-C significantly increased in the MD group (*p* < 0.05) but not in the MD+ONS group. In the LDL fraction, total LDL-P and small LDL-P decreased in the MD group (*p* < 0.05), while medium LDL-P increased across both groups (*p* < 0.01). Total HDL-P decreased (*p* < 0.05), yet large HDL-P significantly increased in the whole cohort and the MD group (*p* < 0.05). No significant changes were observed in structural lipids or total fatty acid families. Conclusions: MD+ONS induces lipidomic shifts that are significantly modulated by baseline sarcopenia. The intervention appears to stabilize VLDL and IDL levels in patients with sarcopenia compared to diet alone, while promoting a more favorable VLDL reduction in individuals without sarcopenia, suggesting that early nutritional support for improving body composition is a critical determinant of the metabolic response to specific interventions in patients with HF.

## 1. Introduction

Heart failure (HF) is increasingly recognized as a systemic syndrome characterized by chronic inflammation, impaired energy metabolism, and progressive skeletal muscle wasting, all of which adversely affect prognosis and quality of life [1,2]. Sarcopenia is present in approximately 20–50% of patients with HF, depending on diagnostic criteria and population characteristics, and independently predicts reduced exercise capacity, frailty, increased hospitalizations, and higher mortality [3,4].

Muscle wasting in HF results from the interplay of neurohormonal activation, anabolic resistance, mitochondrial dysfunction, and enhanced catabolic pathways, ultimately leading to loss of muscle mass and strength [5]. Both randomized and observational studies, irrespective of the assessment technique used, consistently demonstrate that sarcopenia in this context is associated with reduced peak oxygen uptake, worse functional class, and increased all-cause and cardiovascular mortality [6,7,8].

Adherence to a Mediterranean dietary pattern (characterized by high consumption of fruits, vegetables, whole grains, legumes, olive oil, and fish) has been associated with a lower incidence of HF and improved cardiovascular outcomes in large cohort studies and randomized trials [4,9]. Among patients with established HF, greater adherence to the Mediterranean diet correlates with better functional status, lower levels of inflammatory biomarkers, and a reduced risk of adverse clinical events [3]. In addition, omega-3 polyunsaturated fatty acids, frequently administered through enriched oral nutritional supplements, exert pleiotropic effects on inflammation, endothelial function, and cardiac remodeling, and have shown modest benefits on mortality and hospitalization rates in HF trials [10,11]. In older adults and populations at risk of sarcopenia, omega-3 supplementation has also been shown to preserve or improve muscle mass, strength, and physical performance, particularly when combined with adequate protein intake [12,13].

Currently, in patients with chronic heart failure NYHA II–IV, eicosapentaenoic acid and docosahexaenoic acid (EPA+DHA) may be considered as adjunctive therapy to reduce cardiovascular mortality and hospitalizations; specifically, dietary sources (especially fish) are preferred, and if high doses are used, the risk of atrial fibrillation should be monitored (more pronounced with >1–2 g/day) [14]. According to a secondary analysis of the OMEMI trial, greater on-treatment increases in EPA were associated with lower risk of major adverse cardiovascular events and higher risk of new-onset atrial fibrillation [15]. Despite this evidence, data specifically assessing the combined effects of a Mediterranean diet plus omega-3-enriched oral nutritional supplements compared with a Mediterranean diet alone in patients with HF, particularly when stratified by the presence or absence of sarcopenia, remain scarce. Moreover, there are no reports about the effect of nutritional interventions or supplementation on the advanced lipidomic profile in patients with HF. This knowledge gap hampers the development of targeted nutritional interventions aimed at mitigating systemic inflammation, metabolic dysregulation, and muscle wasting in this high-risk population. In this context, we aimed to analyze the specific effect of a Mediterranean diet plus hypercaloric, high-protein, omega-3-enriched oral nutritional supplements on the metabolic lipid profile of patients with HF after a 24-week randomized trial of nutritional support in these patients, and specifically in patients with or without sarcopenia.

## 2. Material and Methods

### 2.1. Patients

This study was approved by the Ethics Committee of Reina Sofía University Hospital (Córdoba, Spain; reference number 5164), initially authorized on 21 October 2021, and subsequently updated on 30 May 2023. The study was conducted in accordance with the Declaration of Helsinki and complied with national and international ethical guidelines. A prospective, open-label design was used, and written informed consent was obtained from all participants prior to inclusion. All individuals received detailed information about the study, and only those who agreed to participate were enrolled.

This is a secondary analysis of a clinical trial. The cohort was originally recruited as part of an open, randomized, controlled clinical trial (ClinicalTrials.gov identifier: NCT05848960, accepted on 17 April 2023 and published on 5 May 2023) [13]. Eligible participants were men and women aged >18 and <85 years, with a left ventricular ejection fraction (LVEF) < 50% and at least one hospital admission for heart failure within the previous six months. *N*-terminal pro–brain natriuretic peptide (NT-proBNP) levels were measured at baseline and at the end of the study. LVEF was assessed by transthoracic echocardiography at the same time points. A blood sample was obtained before 10:00 h in the morning after an 8 h fasting period and analyzed the same day in the hospital laboratory. An additional serum sample for the advanced lipoprotein analysis was stored at −80 °C for simultaneous analysis (of all collected samples) at the end of the study.

### 2.2. Nutritional Support

This was a randomized, open, controlled, clinical trial. Thirty-eight patients were randomly assigned by the clinical investigator to receive either a Mediterranean diet alone or a Mediterranean diet plus two hypercaloric, hyperproteic oral nutritional supplements (ONS) per day with a 1:1 allocation for twenty-four weeks. Patients were recruited between January and July 2021. The baseline visit was considered the first day of clinical evaluation, and the final visit was considered the clinical evaluation after 24 weeks. Four patients died during follow-up in the control group and one patient in the intervention group. The ONS formulation included slow-release carbohydrates, a fiber blend, and a combination of *n*-3 and *n*-6 fatty acids, providing 385 mg of EPA and DHA per 100 mL. The supplements were supplied by Nutrisens^®^. All included patients presented high adherence to the ONS (75%, represented by the intake of at least 1.5 ONS per day or 10.5 ONS per week; for controlling adherence, empty bottles were controlled every 2 weeks). Adherence to the Mediterranean Diet was assessed using the MEDAS14 questionnaire; all patients reported a score > 9 at the end of the study.

At baseline, all participants received standardized education and counseling on nutritional support, adherence to the Mediterranean diet, and physical activity. In addition, all patients received oral calcifediol supplementation. Participants were further classified according to the presence or absence of sarcopenia (no sarcopenia, *n* = 13; sarcopenia, *n* = 25), defined as a handgrip strength below the 3rd percentile adjusted for age and sex. No other parameters were included for the definition of sarcopenia, since body composition analysis might be altered due to water content in these patients and other functional tests can be altered due to dyspnea, which is directly associated with HF.

### 2.3. Advanced Lipoprotein Profile

Frozen samples were shipped to Biosfer Teslab (Reus, Spain) for NMR analysis. Advanced lipoprotein profiling was performed using the Liposcale test [16]. This analysis included triglyceride (TG) and cholesterol (C) concentrations, particle size (Z), and particle concentration (P) of the three major lipoprotein classes [very-low-density lipoprotein (VLDL), low-density lipoprotein (LDL), and high-density lipoprotein (HDL)], as well as particle concentrations of nine subclasses (large, medium, and small VLDL, LDL, and HDL). Briefly, particle concentrations and diffusion coefficients were derived from the amplitudes and attenuation of spectrally distinct lipid methyl group signals obtained by two-dimensional diffusion-ordered ^1^H-Nuclear magnetic resonance (NMR) spectroscopy (DSTE pulse sequence). Briefly, high-resolution ^1^H-NMR spectra were acquired on a Bruker Avance III 600 MHz spectrometer operating at a proton frequency of 600.20 MHz (14.1 T) and 310 K. We used the LED (ledbpgp2s1d) pulse sequence—a double stimulated echo with bipolar gradients and longitudinal eddy current delay—together with 1D NOESY experiments, and Carr–Purcell–Meiboom–Gill (CPMG) were used to characterize small molecules such as amino acids and sugars. The relaxation delay was 2 s, and 64k complex points were collected for each scan. The methyl signal was fitted to nine Lorentzian functions corresponding to each lipoprotein subclass. The area under each Lorentzian curve was proportional to the lipid concentration of the corresponding subclass, while particle size was calculated from the diffusion coefficient. Particle concentrations for each subclass were determined by dividing lipid volume by the particle volume of the respective class. Lipid volumes were estimated using established conversion factors to transform concentration units into volume units [17]. Finally, weighted mean particle sizes for VLDL, LDL, and HDL were calculated by summing the diameter of each subclass weighted by its relative contribution to total particle number [18].

In the results section, data is reported according the following classification: VLDLs (VLDL-C, VLDL-TG, VLDL-P, large VLDL-P, medium VLDL-P, and small VLDL-P); intermediate lipoproteins (IDL: IDL-C, IDL-TG); LDL lipoproteins (LDL-C, LDL-P, large LDL-P, medium LDL-P, small LDL-P, and LDL-TG); and HDLs (HDL-C, HDL-TG, HDL-P, large HDL-P, medium HDL-P, and small HDL-P). LMWMs included structural lipids [Phospholipids (PL), Phosphatidylcholine (PC), Sphingomyelin (SM), Lysophosphatidylcholine (LPC)] and fatty acids [(saturated fatty acids (SFA), polyunsaturated fatty acids (PUFA), linoleic acid (LA), Omega-6/Omega-7 (w6/w7), Omega-9 (w9), docosahexaenoic acid (DHA)].

### 2.4. Statistical Analysis

Between-group comparisons were analyzed by the Mann–Whitney U test (nonparametric data). Paired analysis was performed using the Wilcoxon test (nonparametric data). Comparisons between more than two groups were performed using ANOVA. The chi-squared test was used to compare categorical data. Statistical analyses were performed using SPSS statistical software version 20 and Graph Pad Prism version 6. Due to the small sample size, and to correct for multiple testing across variables, all reported *p*-values were adjusted using the Holm–Bonferroni method. Data are expressed as median with interquartile range and percentages. Absolute differences in some parameters were calculated using mean values. For specific group analysis, the absolute number has also been expressed in brackets. *p*-values < 0.05 were considered statistically significant.

## 3. Results

Thirty-eight patients were included in this clinical trial; 65,78% had sarcopenia (*n* = 25). Changes in metabolites were analyzed depending on the presence of sarcopenia; in this section, only significant results are presented. Baseline clinical characteristics were similar in both groups, except for age, which was significantly higher in the group with sarcopenia (median 72 vs. 64, *p* = 0.02). Baseline characteristics are shown in Table 1.

### 3.1. Clinical Correlations Between Cardiac Function and the Metabolomic Lipid Profile

NT-proBNP levels were positively correlated with ω6/ω7 fatty acids (Spearman’s ρ = 0.435, *p* < 0.05), ω9 fatty acids (ρ = 0.583, *p* < 0.01), and IDL triglycerides (ρ = 0.477, *p* < 0.05) in all patients at baseline. LVEF was not correlated with any of the evaluated metabolomic parameters.

### 3.2. Changes in Lipid Profile

#### 3.2.1. Very-Low-Density Lipoproteins (VLDL)

In the whole cohort, VLDL cholesterol (VLDL-C) significantly decreased over time (*p* < 0.001). VLDL particle size (VLDL-Z), VLDL particle concentration (VLDL-P), and large VLDL also significantly decreased during the 24 weeks of intervention (Table 2).

**Figure 1:**
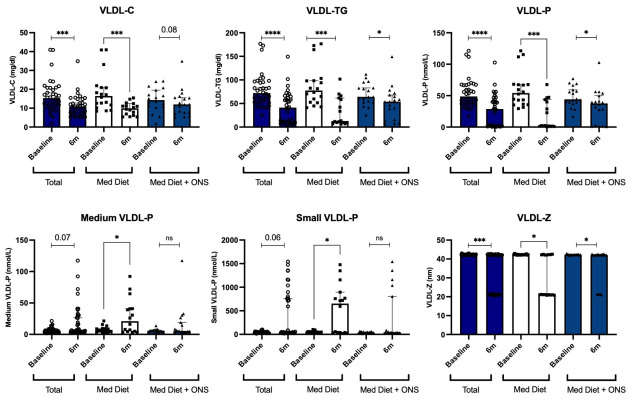
Metabolomic alterations in VLDL subfraction profiles across control and intervention groups in the whole cohort. Legend: VLDL, very-low-density lipoprotein; TG, triglycerides; P, particle concentration; Z, mean particle diameter; ns, non-significant; * *p* < 0.05; *** *p* < 0.001; **** *p* < 0.0001.

A more pronounced reduction in VLDL-C was observed in the control group (*p* < 0.01; Table 3) and a non-significant trend in the intervention group (*p* = 0.08). VLDL triglycerides (VLDL-TG) and VLDL particle concentration (VLDL-P) significantly decreased in the entire cohort (*p* < 0.001), as well as in both the control (*p* < 0.001) and intervention groups (*p* = 0.03; Table 3). Medium and small VLDL-P significantly changed in the control group (*p* < 0.05) but remained unchanged in the intervention group. VLDL-diameter (VLDL-Z) significantly decreased in the whole cohort (*p* < 0.001), particularly in the control group (*p* < 0.05; Table 3; Figure 1).

**Figure 2:**
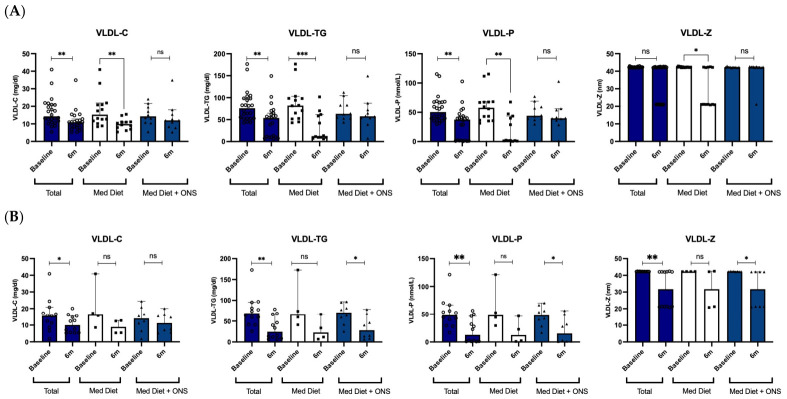
Metabolomic alterations in IDL subfraction profiles across control and intervention groups according to the presence of sarcopenia (**A**) or no sarcopenia (**B**). Legend: VLDL: very-low-density lipoproteins; TG: tryglicerides; P: particle concentration; Z: particle size; ns: non-significant; * *p* < 0.05; ** *p* < 0.01; *** *p* < 0.001.

In patients with sarcopenia, VLDL-C levels were significantly higher than in the control group (*p* = 0.08), while no significant changes were observed in the intervention group. A similar pattern was observed for VLDL-TG (*p* = 0.03) and VLDL-P (*p* = 0.04) (Table 4; Figure 2A).

In patients without sarcopenia, VLDL-C significantly decreased in the whole group (*p* < 0.05), without significant changes when groups were analyzed separately. VLDL-TG, VLDL-P, and VLDL-Z significantly decreased in the whole cohort (*p* < 0.01), driven mainly by significant reductions in the intervention group (*p* < 0.05), with no significant changes in the control group (Table 5; Figure 2B).

#### 3.2.2. Intermediate-Density Lipoproteins

**Figure 3:**
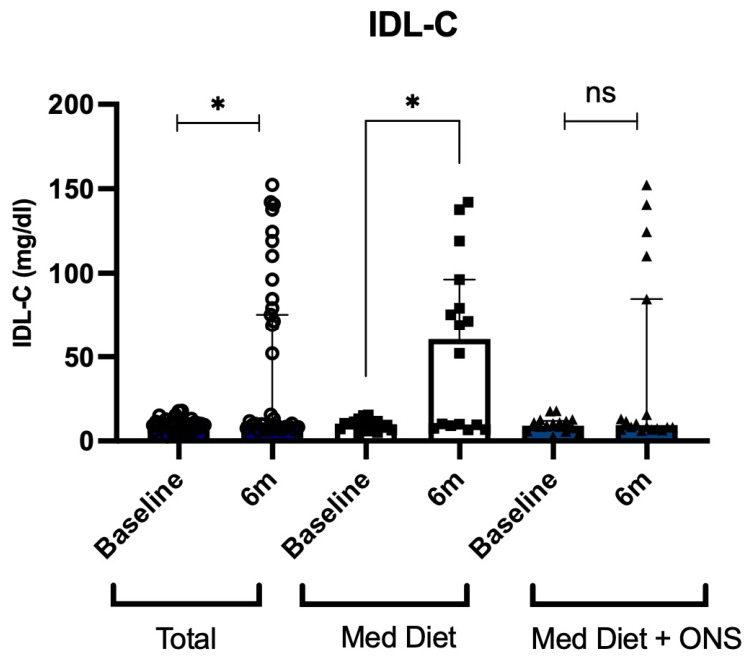
Metabolomic changes in the IDL lipid profile in the control and intervention group. Legend: IDL: intermediate density lipoproteins; ns: non-significant; * *p* < 0.05.

IDL cholesterol (IDL-C) significantly increased in the whole cohort (*p* = 0.037; Table 2) and specifically in the control group (*p* = 0.031), while no significant changes were observed in the intervention group (Table 3; Figure 3). These findings were consistent regardless of the presence or absence of sarcopenia.

#### 3.2.3. Low-Density Lipoproteins

In the whole cohort, LDL particle concentration (LDL-P) significantly decreased (*p* = 0.035), while its diameter and large, medium, and small LDL-P significantly changed over time (*p* < 0.05; Table 2).

**Figure 4:**
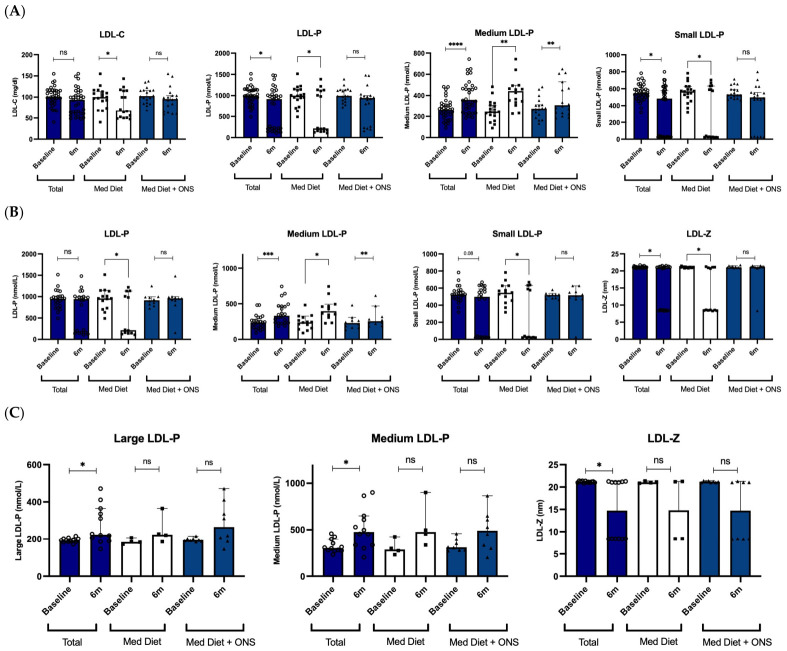
Metabolomic alterations in LDL subfraction profiles across control and intervention cohorts. Comparative analysis of LDL lipidomic shifts in the total study population (**A**), patients with sarcopenia (**B**), and patients without sarcopenia (**C**). Legend: LDL: low density lipoproteins; P: particle concentration; Z: particle size; ns: non-significant; * *p* < 0.05; ** *p* < 0.01; *** *p* < 0.001; **** *p* < 0.0001.

When the nutritional interventions were analyzed, LDL cholesterol (LDL-C) and LDL particle concentration (LDL-P) significantly decreased in the control group (*p* = 0.041) but not in the intervention group (Table 3). Large LDL-P showed a trend toward an increase in the intervention group (*p* = 0.06) and significantly increased in the whole cohort (*p* < 0.05). Medium LDL-P significantly increased in the entire cohort (*p* < 0.001), as well as in both the control (*p* < 0.01) and intervention groups (*p* < 0.01). Small LDL-P significantly decreased in the control group (*p* = 0.012; Table 3; Figure 4A).

Among patients with sarcopenia, LDL-P significantly decreased in the control group (*p* = 0.026). Medium LDL-P significantly increased in the whole cohort (*p* < 0.001), including both the control (*p* = 0.021) and intervention groups (*p* = 0.009). Small LDL-P and LDL particle size (LDL-Z) significantly decreased in the control group (*p* < 0.05), while remaining unchanged in the intervention group (Table 4; Figure 4B).

In patients without sarcopenia, large and medium LDL-P significantly increased in the whole cohort, while LDL-Z significantly decreased (*p* < 0.05), without significant changes when groups were analyzed separately (Table 5; Figure 4C).

#### 3.2.4. High-Density Lipoproteins

In the overall study population, total HDL particle concentration (HDL-P) significantly decreased, as well as its diameter and the large and small HDL-P (*p* < 0.05; Table 2).

**Figure 5:**
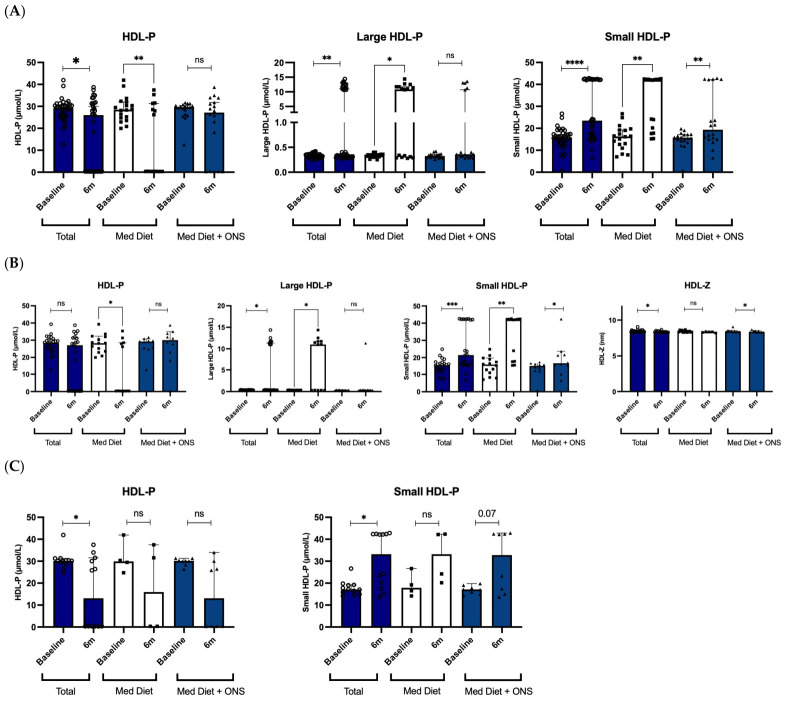
Metabolomic alterations in HDL subfraction profiles across control and intervention groups. Comparative analysis of the total study population (**A**), patients with sarcopenia (**B**), and patients without sarcopenia (**C**). Legend: HDL, high-density lipoprotein; P, particle concentration; Z, mean particle diameter; ns, non-significant; * *p* < 0.05; ** *p* < 0.01; *** *p* < 0.001; **** *p* < 0.0001.

HDL-P decreased particularly in the control group (*p* = 0.08; Table 3). In contrast, large HDL-P (*p* < 0.05) and small HDL-P significantly increased in both the control and intervention groups (*p* < 0.01; Table 3; Figure 5A).

In patients with sarcopenia, HDL-P significantly decreased in the control group (*p* = 0.026). Large HDL-P significantly increased in the whole cohort (*p* < 0.05) and specifically in the control group (*p* < 0.05). Small HDL-P significantly increased in the whole cohort (*p* < 0.01), including in the control group (*p* = 0.038). HDL-P significantly decreased in the intervention group (*p* = 0.036; Table 4; Figure 5B).

In patients without sarcopenia, HDL-P significantly decreased in the whole cohort (*p* < 0.05), without significant changes when groups were analyzed separately. Conversely, small HDL-P significantly increased in the whole cohort (*p* < 0.05), with a trend toward increase in the intervention group (*p* = 0.07; Table 5; Figure 5C).

#### 3.2.5. Structural Lipids and Lipid Families

In the whole cohort, no significant changes were observed in structural lipids (PL, PC, SM, and LPC) or lipid families (SFA, PUFA, LA, ω3, ω6/ω7, ω9, DHA, and ARA-EPA; Table 2). Serum ARA-EPA levels significantly increased in patients who received the Mediterranean diet plus ONS (*p* = 0.03; Table 3). No other significant changes were observed in structural lipids (PL, PC, SM, and LPC) or lipid families (SFA, PUFA, LA, ω3, ω6/ω7, ω9, and DHA) in both groups. When stratified by sarcopenia, no significant changes were observed in structural lipids of lipid families.

### 3.3. Clinical Associations with Sarcopenia and Mortality

During the study period, five patients died. Of these, four presented with sarcopenia and were assigned to the control group, whereas the remaining patient did not present with sarcopenia and was allocated to the intervention group. Mortality was due to a primary thromboembolism in the latter case, while HF was the cause of death in the remaining cases. There were no significant differences in mortality across the groups (*p* = 0.44). The lipid profile was not associated with baseline sarcopenia or mortality in either univariate or multivariable binary logistic regression models adjusted for age and sex in this cohort.

## 4. Discussion

HF patients in this trial exhibited complex changes in NMR-derived lipoprotein subclasses and fatty acids in response to nutritional support, providing novel insight into the interplay among lipid metabolism, sarcopenia, and cardiac biomarkers beyond conventional lipid measurements.

The positive correlations between NT-proBNP and ω-6/ω-7 and ω-9 fatty acids, as well as IDL triglycerides, suggest that more advanced cardiac dysfunction is associated with a pro-atherogenic and potentially pro-inflammatory lipidomic profile in this cohort [19]. Prior interventional studies have primarily evaluated the effects of omega-3 supplementation on NT-proBNP and clinical outcomes, with most reporting neutral or modest effects of *n*-3 fatty acids on NT-proBNP concentrations. These findings indicate that circulating natriuretic peptide levels may not sensitively reflect the metabolic actions of omega-3 fatty acids in chronic cardiovascular disease [20]. In contrast, observational and genetic epidemiology studies have linked specific lipoprotein and fatty acid traits to HF risk, supporting the concept that detailed lipidomic signatures may capture dimensions of myocardial stress not reflected by natriuretic peptides alone [21]. The lack of correlation between left ventricular ejection fraction (LVEF) and lipidomic variables in our study is consistent with prior evidence that NT-proBNP more accurately reflects hemodynamic load and neurohormonal activation, whereas LVEF is an imperfect surrogate of ongoing myocardial stress, particularly in elderly, multimorbid populations with preserved or mildly reduced ejection fraction [22].

In the overall cohort, significant reductions in VLDL cholesterol, triglycerides, and particle concentration, together with decreased VLDL particle size, indicate a shift toward fewer and smaller triglyceride-rich lipoproteins over 24 weeks. Previous NMR-based profiling studies have demonstrated that large and very large VLDL particles are strongly associated with incident HF and other cardiovascular events, suggesting that reducing their burden may improve prognosis [21]. Notably, in our trial, the decline in VLDL-C and VLDL-TG was more pronounced in the control group, whereas the intervention group showed only a non-significant trend. This pattern may reflect the hypercaloric and hyperproteic composition of the oral supplements, which could sustain hepatic VLDL production despite overall nutritional improvement. Similar dissociations between clinical improvement and changes in triglyceride-rich lipoproteins have been described in nutritional and nutraceutical interventions in HF, implying that the benefits of structured nutritional support may be mediated through mechanisms other than VLDL reduction, including enhanced muscle function, attenuation of systemic inflammation, or modulation of cardiac energetics [23].

Stratified analyses according to sarcopenia indicate that body composition substantially modifies the VLDL response to nutritional support. Among patients with sarcopenia, reductions in VLDL-C, VLDL-TG, VLDL-P, and VLDL-Z were largely driven by the control group, whereas the intervention group exhibited minimal changes, suggesting that additional caloric and protein intake may attenuate the decline in triglyceride-rich particles in individuals with advanced muscle wasting. Conversely, in patients without sarcopenia, the principal reductions in VLDL-TG, VLDL-P, and VLDL-Z occurred in the intervention group, indicating a more favorable lipidomic response when baseline muscle mass is preserved and anabolic responsiveness to hyperproteic supplementation is greater [24]. These divergent patterns are consistent with prior studies demonstrating that sarcopenia in HF is associated with altered substrate utilization and reduced capacity to metabolize exogenous nutrients, potentially resulting in distinct patterns of hepatic lipid export and peripheral lipoprotein clearance compared with non-sarcopenic patients [25].

The increase in IDL cholesterol observed in the cohort, particularly in the control group, is noteworthy given the recognized atherogenicity of IDL particles and their association with coronary disease and HF in population studies [21]. The absence of significant changes in IDL-C in the intervention group may suggest a partial protective effect of hypercaloric, hyperproteic supplementation against remnant lipoprotein accumulation, possibly through enhanced hepatic clearance or modulation of lipoprotein lipase activity, although mechanistic inferences cannot be drawn from our data. Prior NMR studies comparing HF patients with healthy controls have reported enrichment in remnant-like lipoproteins, implying that attenuation of IDL-C increases could be beneficial even in the absence of changes in traditional lipid targets such as LDL-C [26].

Regarding LDL subclasses, reductions in LDL-C and total LDL particle concentration in the control group, accompanied by an increase in medium LDL-P and a decrease in small LDL-P, suggest a shift toward a less atherogenic LDL profile over time [27], predominantly among patients receiving only the Mediterranean diet. Population-based NMR analyses have consistently identified small, dense LDL particles as stronger predictors of cardiovascular events than larger LDL subclasses [21]; therefore, the reduction in small LDL-P may reflect favorable remodeling of the LDL fraction in the control arm. The absence of reductions in LDL-C and LDL-P in the intervention group, together with a trend toward increased large LDL-P, may be attributable to the fat content and increased caloric intake associated with supplementation, which can stimulate LDL production despite potential qualitative improvements in fatty acid composition. Comparable neutral effects on LDL-C have been reported in omega-3 and oral nutritional supplement trials in [28]. In participants with sarcopenia, the decline in small LDL-P and LDL particle size observed in the control group but not in the intervention group further supports the hypothesis that metabolic responses to additional caloric intake are attenuated in advanced muscle loss, potentially due to reduced muscular lipid storage capacity and persistent reliance on hepatic export pathways [25].

The observed HDL subclass changes (an overall reduction in total HDL-P with concomitant increases in large and small HDL-P, particularly in the control group) underscore the complexity of HDL remodeling in HF. Genetic and observational studies indicate that medium and large HDL particles, rather than total HDL-C, are inversely associated with HF and other cardiovascular outcomes, whereas very small HDL particles show heterogeneous associations [21]. Nutritional interventions and omega-3 supplementation have variably increased large HDL particles and improved HDL functionality without consistently altering HDL-C, suggesting that qualitative changes in HDL may be more clinically relevant than absolute cholesterol content [29]. In our cohort, the increase in large HDL-P in the overall group and the reduction in HDL particle size among sarcopenic patients receiving supplementation may reflect a dynamic balance among enhanced reverse cholesterol transport, altered triglyceride exchange via cholesteryl ester transfer protein, and changes in hepatic lipase activity under hypercaloric conditions. The absence of significant changes in total phospholipids, sphingomyelins, and ω-3 fatty acids within the structural lipid fraction indicates that the intervention primarily influenced lipoprotein particle number and size rather than substantially modifying the distribution of major lipid classes, which is consistent with other short-term lipidomic studies of nutritional interventions [29].

An additional relevant finding is that the baseline lipidomic profile was not independently associated with mortality in logistic regression models adjusted for age and sex. This may reflect the limited sample size, short follow-up, and universal provision of structured nutritional support, which could have attenuated risk differentials between sarcopenic and non-sarcopenic patients [25]. Large-scale NMR studies have demonstrated that certain lipoprotein subclasses, including XXL-VLDL and medium HDL, predict incident HF and cardiovascular events; however, the prognostic value of these markers in intensively treated, elderly HF cohorts remains uncertain and likely requires substantially larger samples to detect modest effect sizes [21]. Therefore, the absence of a significant association between lipidomic variables and mortality in our study does not exclude a potential etiological role but highlights the challenges of translating advanced metabolomic profiling into clinically actionable risk stratification tools in small interventional trials [29].

This study has several limitations. First, the relatively small sample size may limit the generalizability of the findings and reduce the statistical power to detect differences between groups. Second, detailed caloric intake data at baseline and study completion were not systematically collected. More importantly, it is not possible to establish a direct causal relationship between any specific component of the ONS formulation (e.g., EPA and DHA enrichment, total caloric content, or protein content) and the observed clinical effects. Due to ethical reasons, a placebo arm was not included in this study. The study also has several strengths, including its relatively long duration, contrasting with most nutritional intervention studies in HF, which rarely exceed three months, as well as the high adherence to both ONS and the Mediterranean diet.

## 5. Conclusions

Taken together, these findings indicate that hypercaloric, hyperproteic, and omega-3-enriched oral supplementation in HF is associated with nuanced alterations in lipoprotein subclasses that depend on sarcopenia status and only partially parallel improvements in NT-proBNP, LVEF, and clinical outcomes previously reported in this cohort [13]. The dissociation between favorable clinical effects and neutral or mixed changes in certain atherogenic lipoprotein fractions suggests that the benefits of nutritional support in HF are likely mediated predominantly through improvements in muscle mass, functional capacity, and systemic inflammation rather than through uniform optimization of the lipid profile [23]. Larger studies integrating lipidomics, comprehensive body composition assessment, and hard cardiovascular endpoints are warranted to determine whether specific lipoprotein signatures can identify patients most likely to benefit from nutritional interventions or who may require adjunctive targeted metabolic therapies.

## Figures and Tables

**Table 1:** Baseline clinical characteristics of the patients. Comparison between groups based on the presence of sarcopenia.

Characteristics	Total (*n* = 38)	No Sarcopenia (*n* = 13)	Sarcopenia (*n* = 25)	*p*
Sex (♂/♀)	71.1%/28.9% (11/27)	61.5%/38.5% (8/5)	24//76% (6/19)	0.29
Age (years)	66.7 ± 13	64 (20.5)	72.5 (22)	0.02
Active tobacco exposure (%)	42.10 (7/38)	46.2 (6/13)	40 (10/25)	0.49
Type 2 diabetes (%)	42.1 (16/38)	23.1 (3/13)	52 (13/25)	0.09
Previous ischaemic cardiomyopathy (%)	34.2 (13/38)	15.4 (2/11)	44 (11/25)	0.08
Ejection fraction (%)	38.5 ± 16	31.5 (30.25)	33.5 (21.5)	0.10
NT-proBNP (pg/mL)	5768 ± 6646	4616 (7202)	2692.5 (2990)	0.99
Symptoms				
Weight loss (3 months, %)	55.3 (21/38)	46.2 (6/13)	60 (15/25)	0.32
Weight loss (6 months, %)	28.9	15.4 (2/13)	36 (9/25)	0.17
Food intake (%)				0.27
Soft	7.9 (3/38)	0	12 (3/25)	
Normal	92.1 (35/38)	100 (13/13)	88 (22/25)	
Gastrointestinal symptoms (%)	15.8 (6/38)	15.4 (2/13)	16 (4/25)	0.67
Abdominal pain	10.5 (4/38)	7.7 (1/13)	12 (3/25)	0.58
Nauseas/vomits	5.3 (2/38)	0	8 (2/25)	0.43
Diarrhea	5.3 (2/38)	7.7 (1/13)	4 (1/25)	0.57
Physical activity (%)				
Moderate Physical activity	18.4 (7/38)	23.1 (3/13)	16 (4/25)	0.45
Resting time (hours/day)	8 ± 4	5 (6.25)	6.5 (5.5)	0.15
Quality of life				
Self-rated health score	69 ± 22	77.5 (18.75)	70 (31.25)	0.15
Mortality	13.16 (5/38)	7.69 (1/13)	16 (4/25)	0.43

**Table 2:** Metabolomic changes in the total cohort of patients with heart failure after 6 months of nutritional support.

	Baseline				6 Months		
	Median	IQR		Median	IQR		*p*
VLDL-C (mg/dL)	15.63	11	20.8	10.93	7.16	14.77	<0.001
VLDL-TG (mg/dL)	70.48	52.8	95.86	40.43	9.8	64.58	<0.001
VLDL-P (nM)	48.44	38.22	67.07	14.52	10.55	19.14	<0.001
VLDL-Z (nm)	42.28	42.16	42.38	21.61	17.6	42.13	0.001
LargeVLDL-P (nM)	1.33	1.085	1.69	4.14	1.17	39.98	0.044
MediumVLDL-P (nM)	7.93	4.47	30.65	3.15	1.33	5.84	0.073
SmallVLDL-P (nM)	41.62	32.56	56.15	8.84	5.09	41.98	0.061
IDL-C (mg/dL)	9.91	7.435	11.63	11.76	8.15	84.84	0.037
IDL-TG (mg/dL)	10.42	8.89	12.07	10.77	8.97	12.4	0.729
LDL-C (mg/dL)	101.6	89.01	115.8	93.18	60.62	110.8	0.104
LDL-TG (mg/dL)	12.89	10.09	14.63	12.48	9.55	14.74	0.286
LDL-P (nM)	909.3	206.1	1033	757	38.92	1119	0.035
LDL-Z (nm)	21.08	20.96	21.23	21.14	20.89	42.2	0.002
Large LDL-P (nM)	183.2	153	194.7	783.6	323.5	982.9	0.023
Medium LDL-P (nM)	266.6	210.2	310.6	196.9	167.7	453.2	<0.001
Small LDL-P (nM)	552.9	499.6	610.3	318.1	221.7	493.4	0.021
HDL-C (mg/dL)	61.95	56.74	66	66.16	55.28	71.41	0.331
HDL-TG (mg/dL)	16.84	11.61	35.85	12.17	9.99	14.43	0.104
HDL-P (nM)	29.45	25.65	30.59	31.53	20.39	549.5	0.023
HDL-Z (nm)	8.37	8.32	8.455	8.6	8.35	21.11	0.005
Large HDL-P (μM)	0.33	0.305	0.36	12.71	0.33	28.55	0.005
Medium HDL-P (μM)	12.47	11.73	13.47	11.05	0.33	12.83	0.801
Small HDL-P (μM)	15.91	13.28	17.84	14.62	11.98	24.12	<0.001
SFA (mM)	9.25	8.69	9.83	9.01	8.395	9.63	0.175
PUFA (mM)	13.16	11.73	14.38	13.29	12.17	14.52	0.495
DHA (mM)	0.17	0.15	0.19	0.17	0.16	0.2	0.172
ARA-EPA (mM)	1.54	1.42	1.73	1.65	1.48	1.975	0.155
LA (mM)	3.57	3.16	3.97	3.71	3.095	4.1	0.955
w6w7 (mM)	5.41	4.86	6.08	5.48	4.65	6.63	0.779
w9 (mM)	4.17	3.77	5.05	4	3.305	4.65	0.282
w3 (mM)	0.31	0.25	0.37	0.32	0.28	0.38	0.466
EC (mM)	2.06	1.81	2.38	2.11	1.94	2.565	0.779
FC (mM)	1.69	1.6	1.88	1.74	1.5	1.94	0.601
PL (mM)	2.82	1.41	3.87	3.53	2.82	4.44	0.085
PC (mM)	1.87	1.61	2.06	1.97	1.69	2.265	0.729
SM (mM)	0.84	0.78	0.9	0.87	0.78	0.92	0.734
LPC (mM)	0.56	0.51	0.61	0.55	0.515	0.605	0.731

Legend: VLDL, very-low-density lipoproteins; IDL, intermediate-density lipoproteins; LDL, low-density lipoproteins; HDL, high-density lipoproteins; C, cholesterol; TG, triglyceride; P, particle size; Z, diameter; SFA, saturated fatty acids; PUFA, polyunsaturated fatty acids; DHA, docosahexaenoic acid; ARA, arachidonic acid; EPA, eicosapentaenoic acid; LA, linoleic acid; w, omega; EC, colesterol esters; FC, free colesterol; PL, phospholipids; PC, phosphatidylcholine; SM, sphingomyelin; LPC, lysophosphatidylcholine; mM, milimolar; nm, nanometers; nM, nanomolar; μM, micromolar.

**Table 3:** Metabolomic changes in the total cohort of patients with heart failure after 6 months of nutritional support according to the nutritional intervention.

		Mediterranean Diet						Mediterranean Diet Plus ONS						
	Median	IQR		Median	IQR		*p*	Median	IQR		Median	IQR		*p*
VLDL-C (mg/dL)	16.45	11.3	21.19	10.42	6.94	12.44	0.001	15.11	10.8	19.55	11.98	7.7	15.58	0.084
VLDL-TG (mg/dL)	77.54	52.36	99.53	13.42	9.69	61.66	0.001	64.14	52.87	82.95	53.78	15.53	67.93	0.031
VLDL-P (nM)	54.24	36.17	67.68	2.35	1.59	43.6	0.001	44.91	39.4	59.24	36.21	2.84	49.74	0.028
VLDL-Z (nm)	42.28	42.12	42.41	21.24	21.05	42.31	0.021	42.28	42.19	42.35	42.05	21.39	42.4	0.049
Large VLDL-P (nM)	1.5	1098	1743	1.7	1.18	5.84	0.053	1.31	1.08	1.56	1.46	1.15	2.96	0.423
Medium VLDL-P (nM)	21.07	4.61	40.85	5.36	4.37	9.12	0.036	5.76	3.55	19.00	5.74	4.02	6.86	0.758
Small VLDL-P (nM)	47.19	30.96	57.69	594	38.92	895.2	0.047	37.65	33.44	50.8	46.21	32.52	806.3	0.619
IDL-C (mg/dL)	9.98	6963	11.54	52.13	9.19	96.35	0.031	9.35	7.71	11.85	10.29	7.48	84.84	0.554
IDL-TG (mg/dL)	10.11	8815	11.93	10.01	8.73	12.55	0.865	10.57	9.1	12.11	11.36	9.21	12.16	0.758
LDL-C (mg/dL)	99.98	86.57	113.8	70.42	52.65	115.2	0.041	103.5	87.09	116.2	94.4	66.22	103.8	0.943
LDL-TG (mg/dL)	12.05	9.19	14.79	12.17	10.97	13.31	0.650	12.91	10.87	14.83	11.77	9.19	15.05	0.266
LDL-P (nM)	217.00	149.1	1126.00	1008	947.2	1145	0.017	928.00	263.7	1002	998.7	896.8	1135	0.687
LDL-Z (nm)	21.09	20.9	21.21	8.6	8.4	21.09	0.009	21.06	21.01	21.36	21.04	8.42	21.27	0.127
Large LDL-P (nM)	179.8	146.4	190.00	189.4	143.7	227.1	0.191	185.00	154.6	199.4	189.00	169.4	238.9	0.062
Medium LDL-P (nM)	244.2	194.00	313.3	426.6	334.3	497.2	0.005	279.8	217.6	312.5	318.1	232.8	528.7	0.006
Small LDL-P (nM)	569.9	489.4	627.4	38.83	23.84	631.4	0.012	538.2	498.1	593.4	490.3	31.92	556.8	0.523
HDL-C (mg/dL)	58.53	54.72	66.75	68.41	60.9	80.85	0.125	63.33	58.79	66.04	64.4	53.61	70.35	0.868
HDL-TG (mg/dL)	24.01	11.97	47.73	14.72	12.21	16.4	0.078	15.07	11.54	22.6	13.7	11.45	16.87	0.586
HDL-P (nM)	28.5	24.44	32.07	0.44	0.32	31.32	0.008	29.68	26.42	30.37	26.42	0.37	31.79	0.723
HDL-Z (nm)	8365	8285	8468	8.28	8.25	8.36	0.125	8.38	8.33	8.44	8345	8.28	8443	0.019
Large HDL-P (μM)	0.335	0.31	0.36	10.7	0.3	11.73	0.016	0.33	0.3	0.36	0.36	0.31	10.71	0.117
Medium HDL-P (μM)	12.42	11.66	13.56	12.3	10.69	13.38	0.755	12.47	11.73	13.47	12.88	11.81	14.77	0.906
Small HDL-P (μM)	16.3	12.24	19.35	42.05	17.78	42.31	0.006	15.89	14.27	17.28	21.28	15.13	42.21	0.004
SFA (mM)	9.22	8.74	9.93	8.54	8.19	9.45	0.16	9.26	8485	9813	9015	8678	9665	0.64
PUFA (mM)	13.16	11.43	13.68	13.51	12.29	13.93	0.50	13.05	11.88	14.62	13.1	12.06	15.24	0.83
DHA (mM)	0.17	0.14	0.19	0.17	0.17	0.2	0.171	0.17	0.16	0.19	0.17	0.16	0.2025	0.06
ARA-EPA (mM)	1.5	1.42	1.73	1.62	1.51	1.82	0.334	1.56	1415	1735	1.67	1445	2.06	0.03
LA (mM)	3.58	3.15	3.86	3.73	2.94	4.12	0.69	3.56	3195	4103	3655	3143	4098	0.72
w6w7 (mM)	5.51	4.97	6.02	5.48	4.62	6.25	0.820	5.28	4795	6.32	5595	4613	6.66	0.868
w9 (mM)	4.17	3.77	5.15	3.99	3.04	4.68	0.112	4.05	3688	4.89	4.08	3365	4.81	0.943
w3 (mM)	0.3	0.24	0.36	0.34	0.29	0.36	0.334	0.315	0.29	0.3775	0.31	0.2725	0.3975	0.924
EC (mM)	2.05	1.74	2.23	2.11	2.00	2.29	0.495	2175	1838	2505.00	2125.00	1.76	2.67	0.831
FC (mM)	1.63	1.59	1.84	1.79	1.5	1.93	0.363	1735	1605	2.03	1705.00	1.48	1955	0.210
PL (mM)	2.77	1.22	3.87	3.4	3.05	4.43	0.173	2.86	1753	4.02	3.57	1708	4575	0.277
PC (mM)	1.87	1.53	2.04	1.91	1.74	2.01	0.798	1.9	1.63	2.27	2025	1615	2388	0.831
SM (mM)	0.82	0.77	0.87	0.87	0.78	0.91	0.161	0.855	0.79	0.9725	0.865	0.765	0.935	0.218
LPC (mM)	0.55	0.51	0.6	0.56	0.52	0.6	0.711	0.58	0.4925	0.63	0.545	0.51	0.6125	0.408

Legend: VLDL, very-low-density lipoproteins; IDL, intermediate-density lipoproteins; LDL, low-density lipoproteins; HDL, high-density lipoproteins; C, cholesterol; TG, triglyceride; P, particle size; Z, diameter; SFA, saturated fatty acids; PUFA, polyunsaturated fatty acids; DHA, docosahexaenoic acid; ARA, arachidonic acid; EPA, eicosapentaenoic acid; LA, linoleic acid; w, omega; EC, colesterol esters; FC, free colesterol; PL, phospholipids; PC, phosphatidylcholine; SM, sphingomyelin; LPC, lysophosphatidylcholine; mM, milimolar; nm, nanometers; nM, nanomolar; μM, micromolar.

**Table 4:** Metabolomic changes in the total cohort of patients with heart failure with sarcopenia after 6 months of nutritional support according to the nutritional intervention.

		Mediterranean Diet						Mediterranean Diet Plus ONS						
	Median	IQR		Median	IQR		*p*	Median	IQR		Median	IQR		*p*
VLDL-C (mg/dL)	15.27	11.3	21.19	9.92	7.18	11.14	0.008	14.28	10.72	20.08	12.01	8253	16.05	0.386
VLDL-TG (mg/dL)	81.96	52.36	99.53	12.41	9.6	60.83	0.003	63.16	52.83	88.36	57.58	50.06	76.01	0.386
VLDL-P (nM)	57.72	36.17	67.68	2.07	1275	42.7	0.004	44.11	38.81	61.69	39.76	34.29	54.47	0.285
VLDL-Z (nm)	42.31	42.08	42.46	21.12	21.06	42.28	0.045	42.29	42.04	42.37	42.29	42.04	42.61	0.878
Large VLDL-P (nM)	1.55	1098	1743	2.17	1298	5.62	0.091	1265	1055	1633	1.31	1135	1825	0.767
Medium VLDL-P (nM)	23.37	5145	40.85	6165	4255	9165	0.110	5.49	3353	9935	5635	3.62	6798	0.959
Small VLDL-P (nM)	51.06	30.96	57.69	654	38.93	764.2	0.091	37.33	33.06	51.47	35.3	31.73	57.18	0.721
IDL-C (mg/dL)	9745	6858	11.08	60.56	9305	78.37	0.062	9995	7225	12.72	8205	6.85	13.86	0.445
IDL-TG (mg/dL)	10.11	8815	11.9	10	8725	12.38	0.657	10.78	8975	12.14	11.81	9545	12.6	0.575
LDL-C (mg/dL)	97.33	72.17	111.3	67.47	50.77	111.3	0.062	91	82.96	99.41	97.62	89.92	103.7	0.093
LDL-TG (mg/dL)	12.05	9015	14.19	11.9	9198	13.29	0.722	12.03	10.05	16.62	10.86	9708	13.28	0.721
LDL-P (nM)	214.5	141.9	1100	957.8	723.6	1132	0.026	959.2	883.1	993	915.8	813.3	991.5	0.241
LDL-Z (nm)	21.09	20.9	21.21	8.58	8428	21.04	0.026	21.03	20.92	21.4	21.15	20.97	21.37	0.812
Large LDL-P (nM)	172.9	136.3	190	159.3	136.3	195.8	0.594	165.3	145.9	186.7	179.3	168.6	186.6	0.203
Medium LDL-P (nM)	239.7	153.3	313.3	396.2	317	486.3	0.021	229.7	165.4	277.1	255.4	213.7	355.9	0.009
Small LDL-P (nM)	545	447.5	627.4	32.97	21.25	620.8	0.016	519.5	479.2	529.5	516.1	472.7	574.3	0.508
HDL-C (mg/dL)	57.96	54.42	66.75	66.34	57.5	70.98	0.091	63.06	56.76	66.57	64.91	53.27	70.49	0.333
HDL-TG (mg/dL)	26.22	13.07	47.73	14.82	12.28	17.01	0.131	14.92	11.67	20.3	14	10.9	17.6	0.799
HDL-P (nM)	28.25	23.38	32.07	0.41	0.325	28.4	0.026	29.19	25.3	29.96	30.03	21.84	34.16	0.386
HDL-Z (nm)	8.41	8.29	8518	8.34	8.24	8.37	0.225	8425	8358	8503	8.36	8285	8505	0.044
Large HDL-P (μM)	0.325	0.31	0.35	10.99	0.3025	11.73	0.038	0.335	0.295	0.38	0.36	0.3125	0.4	0.371
Medium HDL-P (μM)	12.61	11.85	13.56	11.69	9325	13.19	0.477	12.7	11.89	15.14	12.83	11.92	14.88	0.646
Small HDL-P (μM)	15.65	10.28	19.21	42	17.65	42.31	0.013	14.9	12.1	16.06	16.5	13.88	22.11	0.037
SFA (mM)	9.22	8.82	9.93	9.03	8.42	9.63	0.328	9655	8.81	9.95	9025	8705	9665	0.333
PUFA (mM)	13.16	10.73	13.51	13.56	12.29	13.93	0.286	13.12	11.78	16.08	13.64	12.43	17.05	0.646
DHA (mM)	0.17	0.14	0.18	0.18	0.17	0.2	0.108	0.17	0.1575	0.1925	0.185	0.17	0.2325	0.406
ARA-EPA (mM)	1.5	1.42	1.73	1.62	1.51	1.82	0.248	1595	1358	1845	1775	1418	2.13	0.308
LA (mM)	3.58	3.15	3.75	3.73	3.34	4.47	0.374	3705	3245	4615	3.89	3.29	4098	0.575
w6w7 (mM)	5.51	4.97	6	5.91	4.68	6.25	0.477	5605	5103	6613	6265	5103	6735	0.721
w9 (mM)	4.24	3.65	5.15	4.48	3.04	4.74	0.248	4645	3868	5503	4.48	3878	6.4	0.878
w3 (mM)	0.3	0.24	0.33	0.34	0.3	0.36	0.328	0.35	0.255	0.43	0.325	0.2725	0.4925	0.878
EC (mM)	2.05	1.61	2.23	2.11	2.01	2.29	0.306	2.41	1715	2668	2.06	1.5	2633	0.573
FC (mM)	1.67	1.62	1.88	1.8	1.53	2	0.476	1775	1.68	2173	1.75	1368	1955	0.139
PL (mM)	2.42	1.19	3.66	3.13	2.71	4.43	0.374	3115	2505	4195	3.57	1218	4575	0.760
PC (mM)	1.79	1.45	2.04	1.97	1.68	2.01	0.859	2.01	1593	2353	1855	1.41	2375	0.445
SM (mM)	0.84	0.78	0.87	0.87	0.78	0.89	0.513	0.875	0.8025	1018	0.85	0.705	0.935	0.169
LPC (mM)	0.55	0.5	0.58	0.56	0.53	0.6	0.421	0.57	0.465	0.665	0.535	0.5	0.6125	0.406

Legend: VLDL, very-low-density lipoproteins; IDL, intermediate-density lipoproteins; LDL, low-density lipoproteins; HDL, high-density lipoproteins; C, cholesterol; TG, triglyceride; P, particle size; Z, diameter; SFA, saturated fatty acids; PUFA, polyunsaturated fatty acids; DHA, docosahexaenoic acid; ARA, arachidonic acid; EPA, eicosapentaenoic acid; LA, linoleic acid; w, omega; EC, colesterol esters; FC, free colesterol; PL, phospholipids; PC, phosphatidylcholine; SM, sphingomyelin; LPC, lysophosphatidylcholine; mM, milimolar; nm, nanometers; nM, nanomolar; μM, micromolar.

**Table 5:** Metabolomic changes in the total cohort of patients with heart failure without sarcopenia after 6 months of nutritional support according to the nutritional intervention.

		Mediterranean Diet						Mediterranean Diet Plus ONS						
	Median	IQR		Median	IQR		*p*	Median	IQR		Median	IQR		*p*
VLDL-C (mg/dL)	16.45	10.39	34.92	8.955	5.38	12.71	0.07	15.63	8.875	18.71	8.96	6.4	15.96	0.128
VLDL-TG (mg/dL)	66.7	45.78	148.1	22.7	8.045	58.21	0.14	66.7	50.74	88.11	22.7	11.08	57.21	0.018
VLDL-P (nM)	48.44	33.08	104.1	12.57	1.218	40.65	0.14	48.44	34.57	61.18	12.57	1.06	40.9	0.028
VLDL-Z (nm)	42.27	42.22	42.28	31.7	20.89	42.38	0.27	42.28	42.22	42.37	31.7	21.34	42.07	0.018
Large VLDL-P (nM)	1.23	0.9375	2.295	2.015	0.69	11.7	0.47	1.31	0.97	1.505	2.02	1	4.165	0.398
Medium VLDL-P (nM)	15.54	3.763	75.66	5.585	3.653	13.36	0.07	15.54	4.14	31.72	5.74	4.235	7.38	0.499
Small VLDL-P (nM)	41.62	28.48	88.49	467.8	24.3	1332	0.27	41.62	29.16	52.21	467.8	35.76	1258	0.398
IDL-C (mg/dL)	10.86	7.255	14.3	53.28	8.148	130.7	0.27	9.35	7.23	10.94	53.28	8.48	132.6	0.128
IDL-TG (mg/dL)	10.37	7.823	14.74	11.57	9.113	13.96	0.72	10.57	9.27	15.17	10.77	8.26	11.81	0.237
LDL-C (mg/dL)	110.7	102.1	123.7	90.58	54.53	136.3	0.47	115.4	111	119.5	83.95	62.16	118.5	0.176
LDL-TG (mg/dL)	13.09	9.44	17.05	12.52	11.19	15.19	1.00	13.08	11.5	14.1	12.52	9.065	16.06	0.176
LDL-P (nM)	660.5	185.6	1317	1118	1003	1219	0.47	660.5	225.5	1141	1119	1113	1146	0.176
LDL-Z (nm)	21.06	20.88	21.31	14.79	8.385	21.23	0.14	21.18	21.02	21.31	14.79	8.375	21.06	0.176
Large LDL-P (nM)	183.7	174.4	201.6	224	194.4	330.4	0.07	192.6	186.6	201.7	224	198.4	373.7	0.176
Medium LDL-P (nM)	285.8	241.8	393.1	476.7	364.8	800.4	0.07	306.3	280.6	376.1	476.7	317.1	629.1	0.091
Small LDL-P (nM)	591.3	577.4	691.3	309.6	25.14	674.9	0.47	593.4	571.4	658.2	309.6	28.96	625.6	0.176
HDL-C (mg/dL)	62.15	55.96	82.57	74.63	47.47	139.4	1.00	63.33	60.94	67.29	57.19	52.72	72.49	0.237
HDL-TG (mg/dL)	20.7	9.653	89.33	13.25	9.85	18.09	0.47	20.7	10.89	36.94	13.35	12.2	15.65	0.499
HDL-P (nM)	29.9	25.92	39.02	15.94	0.3175	35.99	0.14	30.1	29.18	30.82	15.94	0.335	28.22	0.091
HDL-Z (nm)	8.31	8.26	8.368	8.25	8.25	8.25	0.16	8.35	8.305	8.395	8.34	8.225	8.38	0.273
Large HDL-P (μM)	0.36	0.2775	0.39	5.52	0.295	12.21	0.14	0.32	0.295	0.36	5.52	0.3	13.06	0.173
Medium HDL-P (μM)	11.73	10.35	14.33	12.88	11.5	15.23	0.47	12.2	11.65	13.01	12.88	11.44	15.34	0.612
Small HDL-P (μM)	17.87	14.77	24.77	33.18	21.2	42.27	0.27	17.17	15.42	18.45	33.18	16.26	42.39	0.073
SFA (mM)	9.08	8.54	9.92	8.365	8.048	9.208	0.47	8.99	8.473	9.385	8.97	8.473	9.728	0.499
PUFA (mM)	13.22	11.81	15.83	12.95	9.99	15.96	0.72	12.83	11.97	14.46	12.56	11.46	13.92	0.612
DHA (mM)	0.185	0.12	0.19	0.16	0.1425	0.1925	1.00	0.165	0.16	0.1825	0.16	0.15	0.1775	0.865
ARA-EPA (mM)	1.605	1.425	1.935	1.705	1.28	1.988	1.00	1.535	1.425	1.725	1.67	1.475	1.778	0.735
LA (mM)	3.545	2.983	3.905	3.305	2.445	4.03	0.72	3.39	2.905	3.99	3.47	2.928	4.28	1.000
w6w7 (mM)	5.655	4.968	6.193	4.89	3.928	6.475	0.72	4.97	4.75	5.875	4.94	4.458	6.223	0.866
w9 (mM)	4.105	3.838	6.308	3.365	2.66	4.16	0.27	3.915	3.463	4.35	3.51	3.305	4.12	0.735
w3 (mM)	0.32	0.135	0.43	0.32	0.2525	0.395	0.47	0.305	0.29	0.3225	0.305	0.2575	0.365	0.735
EC (mM)	2.075	1.905	2.388	2.105	1.773	2.43	0.72	2.065	1.893	2.373	2.125	1.885	2.735	0.612
FC (mM)	1.595	1.53	1.615	1.645	1.418	1.895	0.72	1.685	1.578	1.828	1.68	1.525	1.96	0.866
PL (mM)	3.865	2.185	4.053	4.34	3.57	4.428	0.07	2.75	1.365	3.96	3.635	3.045	5.45	0.116
PC (mM)	1.925	1.775	2.06	1.88	1.775	2.33	1.00	1.885	1.67	2.02	2.125	1.79	2.403	0.237
SM (mM)	0.785	0.7275	0.8425	0.865	0.7525	0.9325	0.20	0.83	0.7875	0.92	0.865	0.7925	0.9325	0.735
LPC (mM)	0.575	0.52	0.615	0.55	0.45	0.65	0.72	0.58	0.5175	0.615	0.545	0.5125	0.6375	0.799

Legend: VLDL, very-low-density lipoproteins; IDL, intermediate-density lipoproteins; LDL, low-density lipoproteins; HDL, high-density lipoproteins; C, cholesterol; TG, triglyceride; P, particle size; Z, diameter; SFA, saturated fatty acids; PUFA, polyunsaturated fatty acids; DHA, docosahexaenoic acid; ARA, arachidonic acid; EPA, eicosapentaenoic acid; LA, linoleic acid; w, omega; EC, colesterol esters; FC, free colesterol; PL, phospholipids; PC, phosphatidylcholine; SM, sphingomyelin; LPC, lysophosphatidylcholine; mM, milimolar; nm, nanometers; nM, nanomolar; μM, micromolar.

## Data Availability

The original contributions presented in this study are included in the article. Further inquiries can be directed to the corresponding authors.

## Abbreviations

VLDL, very-low-density lipoproteins; IDL, intermediate-density lipoproteins; LDL, low-density lipoproteins; HDL, high-density lipoproteins; C, cholesterol; TG, triglyceride; P, particle size; Z, diameter; SFA, saturated fatty acids; PUFA, polyunsaturated fatty acids; DHA, docosahexaenoic acid; ARA, arachidonic acid; EPA, eicosapentaenoic acid; LA, linoleic acid; w, omega; EC, colesterol esters; FC, free colesterol; PL, phospholipids; PC, phosphatidylcholine; SM, sphingomyelin; LPC, Lysophosphatidylcholine; mM, milimolar; nm, nanometers; nM, nanomolar; μM, micromolar.
